# Human blood platelets and viruses: defense mechanism and role in the removal of viral pathogens

**DOI:** 10.1186/s12959-018-0170-8

**Published:** 2018-07-17

**Authors:** Masresha Seyoum, Bamlaku Enawgaw, Mulugeta Melku

**Affiliations:** 1 University of Gondar hospital, College of Medicine and Health Sciences, University of Gondar, Gondar, Ethiopia; 2 Department of Hematology & Immunohematology, School of Biomedical and Laboratory Sciences, College of Medicine and Health Sciences, University of Gondar, Gondar, Ethiopia

**Keywords:** Platelets, Viral infections, Platelet activations, Platlet granules, Viral defense mechanisms

## Abstract

Platelets are small non-nucleated cell fragments and the second most abundant cell that play crucial role in managing vascular integrity and regulating hemostasis. Recent finding shows, beyond its hemostatic function platelets also play a main role in fighting against pathogen including viruses. With their receptors, platelet interacts with viral pathogen and this interaction between platelets and viral pathogens result in activation of platelets. Activated platelet releases different molecules that have antiviral activity including kinocidins and other platelet microbicidal peptides. In addition, activated platelet has antiviral role by different mechanism including; phagocytosis of viral pathogen, produce reactive oxygen species and interact with and activate other immune cells. In other side, antiplatelet treatments are one of defending mechanism of viral pathogen. This narrative review summarizes what is known regarding the role of human platelets in fighting viral pathogen.

## Background

Platelets are small discoid shaped non-nucleated cells with 1-3 μm size in diameter. They originated from bone marrow megakaryocytes (MKs) cytoplasm and circulate in the vascular system to play a main role in haemostasis and thrombosis. In normal healthy individual, platelets are the second most abundant cells next to red blood cells (RBCs) with 150,000 – 450,000 cells/μL [1–3]. These number account about 70% of the platelets in the peripheral blood. The remaining 30% body platelets are stored in the spleen [3].

Platelets have a main function in hemostasis and blood coagulation. They involve in the prevention of bleeding disorders, mainly in the primary hemostasis. Following vascular injury, sub endothelial collagen is exposed. Then plasma von Willebrand factor (vWF), which is secreted by platelets and endothelial cells, anchor onto the collagen. Platelet vWF receptor (glycoprotein Ibα) interacts with vWF and initiates platelets to the injured site [4]. Then they became activated and release different molecules such as von Willebrand factor (vWF), thromboxane A2, phospholipids, ADP and others [5]. ADP involved in attraction of more platelets to the injured area and thromboxane A_2_ promotes platelet aggregation to form platelet plug. This platelet plug minimizes bleeding and promote blood coagulation process [4].

Now a day, different evidence showed that platelets have an ability to interact with and internalize pathogens including viruses [6–9], beyond their main function in hemostasis and blood coagulation [4, 5]. These functions are achieved through direct interaction with leukocytes, endothelial cells and via release of soluble inflammatory mediators that enhance recruitment and activation of leukocytes [6, 10–14].

In addition to binding function, platelets also involve in phagocytosis by enhancing antigen presentation by antigen presenting cells [15–18]. But their role in viral infection response is not fully understood. Their interaction to viruses may have benefit or risk for the host. Therefore, this narrative review aimed to summarize the current knowledge on the role of blood platelets in fighting viral pathogens. This review focused on new and emerging concepts related to defending role and mechanism of platelets against viral infections.

### Mechanism of human platelets in defending viral pathogen

On their surface platelets have receptors for viral pathogens that facilitate the direct interaction of platelets with viral pathogens. This interaction between platelets and viral pathogens through different receptors cause quantitative (thrombocytopenia) and qualitative platelets dysfunction associated with viral pathogen [6, 19–23]. Beside this, the recent findings indicate that platelets play a main role in defending viral pathogen and has adverse effect against viral infection. It shows that binding of viral pathogen with platelets not only result in clearance of platelets but also clearance of viral pathogens caused by platelets [6, 7].

### Platelet receptors to recognize viral pathogens

Different receptors that found on the surface of platelets enable platelets to sense the presence of viral pathogens [24]. Platelets and megakaryocytes express messenger ribonucleic acid (mRNA) and/or protein for the toll like receptors (TLR 1, TLR 2, TLR3, TLR 4, TLR 6, TLR 7, TLR 8 and TLR 9) that detect and bind viral components at the cell surface and viral nucleic acids [9, 25–32]. These TLRs are important for binding of platelets to pathogens such as bacteria, parasites and protozoa and important for their clearance [8, 9, 13, 30, 33].

Also, platelets express several complement receptors, including CR2, CR3, CR4, C3aR, C5aR, gC1qR and cC1qR [34]. These complements act as receptor for pathogens and implements multiple functions in direct and indirect antimicrobial host defense, including, cell lysis, opsonization and chemotaxis [35]. Megakaryocytes express CD4 [36] while both MKs and platelets express CXCR1, 2, 4 and CCR3 co receptors used for interaction with HIV-1 [17, 37].

Additionally, platelet granules express Dendritic Cell-Specific ICAM3-Grabbing Non-integrin (DC-SIGN) [18, 38] and C-type lectin receptor 2 (CLEC-2) ([18, 39])which are used for the interaction with HIV-1 [18, 38] Platelet surface receptors such as DC-SIGN or enhancement of the receptor FcγII play important role in binding of platelets with Dengue virus (DENV). DC-SIGN and heparin sulphate proteoglycan are receptors for DENV and the combination of anti–DC-SIGN and low-molecular-wType equation here.eight heparin prevent binding of platelets with DENV [19, 22]. DENV infection leads to thrombocytopenia by increasing phagocytosis of DV-induced apoptosis platelets by macrophages via a Phosphatidylserine-recognizing pathway [23, 40].

Among the receptors, alpha v beta 3 (αvβ3) or alpha v beta 5 (αvβ5) interact with adenoviruses [41] and platelet glycol proteins GPIa/IIa (α2 β1 integrin) and GPVI (a member of the Ig super family and primary signalling receptor for platelet activation by collagen) bind viruses [42]. Platelets also express Coxsackie-Adeno receptor (CAR). This receptor is important for the interaction of Coxsackieviruses B (CVB) with platelets. Although CVB virus does not replicate in platelets, it triggers P-selectin and Phosphatidylserine (PS) membrane expression. Expression of P-selectin and PS as the main mediators of platelet with leukocyte interactions and promoting their phagocytosis by macrophages [43].

Although, platelets do not express HCV receptors such as CD81 [44]; they express glycoprotein VI (GPVI) which is a receptor used to form interaction between HCV and platelets [45]. Studies demonstrated that individuals with HPA-1a/1b, HPA-2b and HPA-5b alleles had a higher risk for HCV than those individuals with HPA-2a and HPA -5b alleles [46–48]. Table 1 summarizes different viruses and their receptors on the platelets.

**Table 1:** Platelet receptors for viruses

Receptor	Viruses	Reference
α_v_β_3_	Hantaviruses	[6, 73, 74]
	Coxsackievirus A9, A16,	
	Human adenovirus type 2	
	Echovirus 9	
	Human parechovirus	
α_IIb_β_3_	Hantaviruses	[6, 73, 74]
α _2_β_1_	Echovirus 1	[75, 76]
	Rotavirus	
DC-SIGN	Lentiviruse, HIV	[9, 38]
DC-SIGN	Ebola virus	[77, 78]
Axl	Lassa fever virus (LASV)	
Tyro3		
CCR-3 & 4, CXCR-1, 2 & 4, CLEC-2, DC-SIGN	HIV	[7, 18]
GP-VI	HCV	[7, 9]
CR2	EBV	[6, 7, 79]
α_2_β_1_	Rotavirus	[7, 9]
α_2b_β_3_ (GP_IIbIIIa_)	Adenovirus	[6, 7]

**N.B:** CLEC-2: C-type lectin-like type II transmembrane receptor; CR-2: Complement Receptor type 2; CCR-1, CCR-3, CCR-4: C-C Chemokine Receptor type 1, 3 and 4; CXCR-1, CXCR-2, CXCR-4: C-X-C chemokine receptor type 1, 2 and 4. DC-SIGN: Dendritic Cell-Specific Intercellular adhesion molecule-3-Grabbing Non-integrin; GP-VI: Glycoprotein VI; PAR-1/PAR-4: Platelet Activating Receptor ¼

### Pro-inflammatory activity of platelets

After platelets bind and phagocytes infectious microorganism, they become activated and undergo degranulation and release a variety of inflammatory mediators, chemokines and cytokines from their granules. Adenosine diphosphate (ADP), thromboxane A2 (TXA2), serotonin, cytokines (IL-1β, TNFα, TGF- β, CD40L) and chemokines (CXCL1 (GROa), CXCL4/PF4, CXCL5, CXCL7, CXCL12, CXCL14, CCL5 (RANTES), CCL3 (MIP1a), MCP-3 (CCL7)) are released. These platelet-derived chemokines and cytokines *stimulate migration of monocytes and* enhances phagocytosis [13, 14, 49–51]*.*

Platelet dense (δ) granules contain nucleotides (ADP and GTP); bioactive amines (histamine and serotonin); and bioactive ions (Ca2+ and PO3−) while alpha (α) granules contain adhesion molecules; platelet microbicidal proteins (PMPs) and kinocidins. The releasing of α - granules provide platelet-mediated host defense mechanisms, as they contain kinocidins and microbicidal proteins which is important for antiviral host defense [50].

Also, when there was infection, platelets play a main role in defending pathogens by activating immune cells. They send signals to immune cells by releasing chemokines that attract and recruit immune cells to the site of infection [52, 53]. For this function, kinocidins, in addition to its antimicrobial activity, play the main role in attraction and activation of phagocytes and lymphocytes [54].

The cleaving of kinocidins and antimicrobial peptides by thrombin and pathogens; give broad and strong antimicrobial property for platelets. For example, the common kinocidins, platelet factor 4 (PF-4) which is a small cytokine belonging to the CXC family, CXCL4, are inhibitor of HIV-1 and suppresses HIV-1 infection of T-lymphocytes. [55–57]. Similar to granulocytes, platelet have direct antimicrobial functions that are mediated by secretion of antimicrobial molecules such as platelet microbicidal peptides (PMPs). PMPs are stored in platelet granules and can kill microorganisms including viruses, when they released during platelet activation and degranulation [58]. Forexample, platelets show a direct interaction with HIV-1 through different mechanisms such as binding, engulfment, and internalization, all of which play a role in host defense during HIV-1 infection, by limiting viral spread and probably by inactivating viral particles [59].

Different observation gives evidence for antiviral activity of antimicrobial peptides. Nine synthetic antimicrobial peptides, four originating from thrombin-induced human platelet-derived antimicrobial proteins named PD1-PD4 and five synthetic repeats of arginine-tryptophan repeats (RW1-5) demonstrate for their antiviral activity for vaccinia virus (VV) [60].

Up on activation platelets also release defensin (α and β) which have anti-viral role. For example, human alpha-defensins 1-3 and human alpha-defensin 5 are potent antagonists of papilloma virus types and human beta-defensins 1 and 2 displayed little anti- human papilloma virus activity [61]*.*

### Platelet ROS production

Reactive Oxygen Species (ROS) are diffusible and short-lived molecules that have a major role in platelet activation regulation. In turn, different observations clearly indicate that, activated platelets have the capability to synthesize ROS such as H_2_O_2_, ^_^OH and peroxynitrite (NO^−^ _3_), which are able to modify platelet function [62, 63]. The released ROS from platelets has antimicrobial role and contribute in defending role of platelets by killing of pathogens [64]. It is widely accepted that the ROS produced by phagocytes during respiratory burst contribute to the elimination of pathogens. For example, recent experiments demonstrated that ROS inhibit human cytomegalovirus (HCMV) infection [65].

### Platelet phagocyte function

Accumulating evidence suggests that, during direct interaction of viral pathogens with human platelets, there is a phagocytosis of viral pathogens by platelets and so enables their transport for a prolonged time. During viral infections there will be expression of P-selectin and PS, which increases platelet-leukocyte interactions and promoting phagocytosis of platelets and viruses by macrophages [23, 43].

HIV is best example to show phagocytosis activity of platelets and conducted research showing that, in platelets incubated with HIV; HIV antigen was found in engulfing vacuoles and the open canalicular system (OCS) and also Immunogold labelling for the viral core protein p24 confirmed the presence of HIV within platelets, which is also true in vivo [66].

Another finding also support phagocytosis role of platelets and platelet suspension incubated with influenza virus were observed by electron microscope, and finding shows that platelets found with virus containing vacuoles, which suggesting that the platelets had rapidly phagocytosed the viruses. Phagocytosis of influenza virus by platelets may play an important role in the occurrence of thrombocytopenia during influenza infection and may also a mechanism of virus clearance during infection [67]. Similar finding is observed in the role of platelets in the clearance of respiratory syncytial virus (RSV) by internalizing viral particles and by enhancing type I IFN production from peripheral blood mononuclear cells [68].

### Platelets interaction with other immune cells

In addition of direct interaction with pathogens and secretion of antimicrobial agents, platelets also interact with other immune cells. After activation platelets secrete a number of chemokines attracting neutrophils as well as cytokines CD40L and IL-1β which inflame endothelial cells. Inflamed endothelial cells express an array of adhesion molecules, such as ICAM-1, VCAM-1, P and E-selectin, promoting neutrophils recruitment [52]. P-Selectin interacts with P -selectin glycoprotein ligand-1 (PSGL-1) and expressed on the surface of leukocytes. This interaction cause, leukocyte mediated immune responses during viral infections by phagocytosis and enhanced reactive oxygen production of neutrophils [69].

Interaction of CD40L on T cells with CD40 on B cells is of paramount importance for the development and function of the humoral immune system. Recent report also indicates that platelets express CD40L when they are activated and interact with CD40 of leukocytes and increases reactive oxygen species (ROS) production by neutrophils which enhance their capacity to phagocytose pathogens [17, 52].

Human cytomegalovirus (HCMV) bind to platelet receptor, TLR 2, that are found on platelet which causes activation and degranulation of HCMV-activated platelet and cause platelets binding to and activate neutrophils results in neutrophils mediated immune response [17]. Also in patients infected with dengue virus, there is direct interaction between platelets and antigen which activate platelets. These activated platelets bind with monocytes and help to enhance immune response for pathogen [70].

Platelets have antigen presenting role [17]. Demonstrated evidences shows that, platelets express T cell costimulatory molecules; process and present antigen in MHC class I, and directly activate naive T cells in a platelet MHC class I-dependent manner. Platelets present pathogen derived antigen to promote T cell responses in vivo; which proofs the novel antigen presentation role of platelets [71]. In a murine model of lymphocytic choriomeningitis virus (LCMV) infection, platelets prevented lethal hemorrhagic anemia by promoting cytotoxic T lymphocyte (CTL) dependent clearance of the virus [72].

## Conclusion

Beyond their primary role in hemostasis, platelets also play a main role in defending viral pathogens. Much more experimental studies give evidence for the antiviral role of platelet and clearly show the binding capacity of platelets with different viral pathogens with their receptors. Platelets activated and initiate their antimicrobial host defense by sensing the presence of pathogens or inflammation through immune receptors such as immunoglobulin or complement receptors and TLRs. After activation platelets secrete a number of chemokines that attract other immune cells which is contribute for the clearance of viruses. Platelet has direct antimicrobial functions that are mediated by secretion of antimicrobial molecules including platelet microbicidal peptides and kinocidins. This antimicrobial peptides releases during platelets activation and kill viral pathogens. The other antiviral role of platelets is killing of viruses by phagocytosis. It is widely accepted that the ROS produced by phagocytes during respiratory burst contribute to the elimination of pathogens. Platelets also produce ROS upon activation which inhibit viruses.

## Abbreviations

ADP: Adenosine diphosphate
ATP: Adenosine triphosphate
C1q: First complement component
CD: Cluster of differentiation
CD40L: CD40 Ligand
CLEC-2: C-type lectin receptor 2
CXCL4: Chemokine (C-X-C motif) ligand 4, or platelet factor 4 (PF4)
DC-SIGN: Dendritic cell-specific ICAM-grabbing non-integrin
DENV: Dengue virus
FcγRІІa: IgG FcγІІa region receptor
HCMV: Human cytomegalovirus
HCV: Hepatitis C virus
HIV: Human immunodeficiency virus
Ig: Immunoglobulin
IL: Interleukin
PAR: Protease-activated receptor
PF4: Platelet factor 4
PMPs: Platelet microbicidal proteins
ROS: Reactive oxygen species
TLR: Toll-like receptor
TNF: Tumour necrosis factor
vWF: von Willebrand factor
